# Overexpression and Biochemical Properties of a GH46 Chitosanase From Marine *Streptomyces hygroscopicus* R1 Suitable for Chitosan Oligosaccharides Preparation

**DOI:** 10.3389/fmicb.2021.816845

**Published:** 2022-01-31

**Authors:** Jianrong Wang, Ping Wang, Mujin Zhu, Wei Chen, Si Yu, Bin Zhong

**Affiliations:** ^1^Shenzhen Raink Ecology & Environment Co., Ltd., Shenzhen, China; ^2^School of Food Science and Engineering, South China University of Technology, Guangzhou, China

**Keywords:** *Streptomyces hygroscopicus* R1, chitosanase, *Pichia pastoris*, chitosan oligosaccharides, overexpression

## Abstract

Due to the various biological activities of chitosan oligosaccharides (COSs), they have great potential value for use in many areas. Chitosanase plays an important role in enzymatic preparation of COSs. Herein, a gene encoding a chitosanase (*Sh*Csn46) from marine *Streptomyces hygroscopicus* R1 was cloned and the sequences encoding *Sh*Csn46 without signal peptide were optimized based on the codon usage of *Pichia pastoris* (*P. pastoris*). In addition, the optimized gene was ligated to pPICZαA and transformed to *P. pastoris* X33. After screening, a recombinant strain named X33-Sh33 with the highest activity was isolated from 96 recombinant colonies. The maximum activity and total protein concentration of the recombinant strain *Sh*Csn46 were 2250 U/ml and 3.98 g/l, respectively. The optimal pH and temperature of purified *Sh*Csn46 were 5.5 and 55°C, respectively. Meanwhile, *Sh*Csn46 was stable from pH 5.0 to 10.0 and 40 to 55°C, respectively. The purified *Sh*Csn46 was activated by Mn^2+^ and inhibited by Cu^2+^, Fe^2+^, and Al^3+^. In addition, substrate specificity of the purified *Sh*Csn46 showed highest activity toward colloidal chitosan with 95% degree of deacetylation. Furthermore, the purified *Sh*Csn46 exhibited high efficiency to hydrolyze 4% colloidal chitosan to prepare COSs. COSs with degree of polymerization of 2–6, 2–5, and 2–4 were controllably produced by adjusting the reaction time. This study provides an excellent chitosanase for the controllable preparation of COSs with a desirable degree of polymerization.

## Introduction

As the second most abundant biopolymer after cellulose in the world, chitin is composed of repeated β-1, 4-*N*-acetylglucosamine (GlcNAc) and mainly from fungal cell walls, exoskeletons of insects, and shells of crustaceans (Kaczmarek et al., 2019). Due to the highly ordered crystalline structure and lack of solubility in organic solvents and water, the industrial application of chitin is limited (Kaczmarek et al., 2019). As the *N*-deacetylated product of chitin, chitosan is soluble in diluted acid and exhibits some practical advantages over chitin (Nayak et al., 2021). Although the solubility of chitosan is improved, high viscosity and poor water solubility limit its further application. Chitosan oligosaccharides (COSs) are derivative products from chitosan or chitin, which exhibit many practical advantages such as high water solubility, low viscosity, biodegradability, and biocompatibility (Sinha et al., 2016; Yuan et al., 2019). Many previous studies revealed that COSs display various biological activities such as being antioxidant, antitumor, antiviral, and antimicrobial (Romanazzi et al., 2018; Zhang et al., 2018; Wu et al., 2019). Furthermore, the biological activities of COSs are related to degree of polymerization (DP), degree of acetylation (DA), and pattern of acetylation (PA) (Hao et al., 2021). The methods for preparation of COSs are mainly composed of the chemical, physical, and enzymatic method. In recent years, the enzymatic method has received more attention for its high specificity, environmental friendliness, and controllability on the final product (Yuan et al., 2019). Although chitin can be used as substrate for enzymatic preparation of COSs, the long time-consuming process and poor efficiency limit its further application. Nowadays, chitosan with different degree of deacetylation (DDA) is the main material for enzymatic preparation of COSs. Enzymatic preparation of COSs from chitosan can be carried out by using non-specific enzymes such as protease and cellulase, and specific enzymes such as chitinase and chitosanase (Kaczmarek et al., 2019). Compared with non-specific enzymes, chitosanase displays higher efficiency and is more suitable for preparation of COSs (Cui et al., 2021).

As a kind of glycoside hydrolase (GH), chitosanase can catalyze hydrolysis of β-1,4 glycoside bonds of chitosan to produce COSs. Chitosanases are produced in many organisms, and mainly from bacteria and fungi (Thadathil and Velappan, 2014). According to the sequences similarity, chitosanase is divided into six GH families (GH5, GH7, GH8, GH46, GH75, and GH80) by CAZy database. Previous research has shown that chitosanases from GH46 have been characterized extensively (Viens et al., 2015). Analysis of the phylogenetic distribution of GH46 members found that the GH46 chitosanases are essentially grouped into five clusters, named A to E (Viens et al., 2015). Furthermore, the characterized GH46 chitosanases are mainly from bacterial, especially from *Streptomyces* and *Bacillus*. Until now, some chitosanases from *Streptomyces* have been reported and characterized (Viens et al., 2015). Most of the reported *Streptomyces* chitosanases exhibit great potential to prepare COSs (Jiang et al., 2012; Sinha et al., 2012; Chen et al., 2021; Guo et al., 2021). It has been reported that the production level of *Streptomyces* chitosanases in wild-type strain are far beneath what is needed for industrial scale application (Thadathil and Velappan, 2014). Therefore, improvement of the production of *Streptomyces* chitosanases is a key factor for further industrial application. Heterelogous expression of chitosanases in *Pichia pastoris* is an effective way to enhance the production of target chitosanases (Peng et al., 2013; Ding et al., 2019; Luo et al., 2020; Wang J. R. et al., 2021). As a mature host, *P. pastoris* is widely used to produce recombinant protein due to its advantages such as extracellular secretion of recombinant protein, low secretion level of endogenous protein, high expression level of target recombinant protein, and mature fermentation process (Ahmad et al., 2014). Therefore, overexpression of target chitosanase in *P. pastoris* could reduce its cost and provide the foundation for its further application.

In this study, a GH46 chitosanase (named *Sh*Csn46) from marine *Streptomyces hygroscopicus* R1 (*S. hygroscopicus* R1) was overexpressed in *P. pastoris* X33 and characterized. Meanwhile, the hydrolytic pattern and preparation of COSs were also investigated. The results of this study will provide an effective method to produce recombinant *Sh*Csn46 and lay a foundation for its application in COS preparation.

## Materials and Methods

### Materials

The *S. hygroscopicus* R1 was isolated from shrimp shell waste and conserved in our laboratory. The *Escherichia coli* (*E. coli*) strain Top 10 competent cell was purchased from Huinuo Biotechnology (Shenzhen, China). The *P. pastoris* X33 and vector pPICZαA were purchased from Invitrogen (Carlsbad, CA, United States).

The T4-DNA ligase, DNA polymerase (PrimeSTAR HS), and restriction enzymes (*Eco*RI, *Not*I, and *Sac*I) were purchased from Takara Biotechnology (Beijing, China). The kits for isolation of plasmid and bacteria DNA were purchased from Tiangen Biotech (Beijing, China). Powdery chitosan with 85, 90, and 95% degree of deacetylation (DDA), glucosamine, microcrystalline cellulose, chitin, xylan, and soluble starch were purchased from Yuanye Biotechnology (Shanghai, China). Chitobiose, chitotriose, chitotetraose, chitopentaose, and chitohexaose were obtained from Long Dragon Biotechnology (Huizhou, China). Furthermore, GlcN, (GlcN)_2_, (GlcN)_3_, (GlcN)_4_, (GlcN)_5_, and (GlcN)_6_ were short for glucosamine, chitobiose, chitotriose, chitotetraose, chitopentaose, and chitohexaose, respectively.

Media for *E. coli* was LBZ (LB with 25 μg/ml zeocin). *P. pastoris* X33 competent cells transformed with expression vectors were screened by YPDZ plates (yeast extract peptone dextrose medium with 100 μg/ml zeocin). BMGY (buffered glycerol complex medium) and BMMY (buffered methanol complex medium) were used to screen the recombinant colonies with higher activities. Media for high cell density fermentation was BSM. LBZ, YPDZ, BMGY, BMMY, and BSM were prepared according to the protocol provided by Invitrogen^1^.

### Gene Cloning and Bioinformatics Analysis

Genomic DNA from *S. hygroscopicus* R1 was extracted using the bacteria genomic DNA extraction kit (Tiangen Biotech, China). Two pairs of primers (sh1717-fw, sh1717-rev and sh201-fw, sh201-rev) were designed for PCR amplification. The primers sh1717-fw (5′-GTGGTGTACGCAGACCGCGAA-3′) and sh1717-rev (5′-TCAGCCGATGTGGTAGTGTC-3′) were designed based on a hypothetical chitosanase sequence from *S. hygroscopicus* strain KCTC1717 (GenBank: CP013219.1, 620221 to 621078, sequence range coding for the hypothetical chitosanase). The primers sh201-fw (5′-GGGCTTCTTTGTCGAATGGTG-3′) and sh201-rev (5′-TATTTCCGCTCCATCCGCATC-3′) were designed based on other hypothetical chitosanase sequences from *S. hygroscopicus* strain XM201 chromosome (GenBank: CP018627.1, 3191731 to 3192597, sequence range coding for the hypothetical chitosanase). The cloned fragment was purified and sequenced. Sequence analysis was carried out by BLASTn and BLASTx provided by the National Center for Biotechnology Information (NCBI). Sequence identity of *Sh*Csn46 against different chitosanases was performed by DNAman 6.0. The signal peptide was analyzed by SignalP 5.0 server. Homology modeling and molecular docking of *Sh*Csn46 were performed by SWISS-MODEL and Autodock vina, respectively. PyMOL was used to analyze the obtained model.

### Overexpression of *Sh*Csn46 in *Pichia pastoris* X33

The gene (named *shcsn46s*) without signal sequence was optimized according to the preferred codons of *P. pastoris*. The optimized gene (*shcsn46s-opt*) flanked with his-tag coding sequence was synthesized by General Biotechnology (Chuzhou, China). The synthetic gene was digested by *Eco*RI and *Not*I and then ligated into pPICZαA to form pPICZαA-*shcsn46s-opt*. The expression vector pPICZαA-*shcsn46s-opt* was linearized with *Sac*I and electrotransformed into *P. pastoris* X33 competent cell. Transformants were plated on YPDZ plates loaded with different zeocin content (from 100 to 300 μg/ml). The method for screening transformants was the same as the previously described method (Wang et al., 2019). The detailed protocol is provided in Supplementary Material.

The recombinant strain with the highest activity was further cultivated in a 7-L bioreactor. The medium composition and cultivation conditions of high cell density fermentation are provided in Supplementary Material. The enzyme activity, wet cell weight, and total protein concentration were monitored throughout the fermentation. The chitosanase activity was measured according to the previous method (Guo et al., 2019). Chitosan (0.5 g) with 95% DDA was dissolved in 100 ml HAc-NaAc buffer (pH 5.5, 0.2 mM) and used as substrate. After 2 min of preheating at 55°C, 50 μl diluted enzyme was added to 350 μl 0.5% (w/v) colloidal chitosan. In addition, the reaction mixture was incubated at 55°C for 10 min, and then 600 μl 3,5-dinitrosalicylic acid (DNS) was added to end the reaction. The reducing sugars released from the substrates were determined with DNS method. One unit of enzyme activity was defined as the amount of enzyme that releases 1 μmol reducing sugars per minute. The concentration of total protein was detected by Bradford method using BSA as standard. Wet cell weight (WCW) was obtained by centrifuging 10-ml samples in a pre-weighted centrifuge tube at 8,000 × *g* for 10 min and discarding the supernatant. SDS-PAGE was used to analyze the supernatant from different induction times.

To further improve the expression level of recombinant *Sh*Csn46 in *P. pastoris*, the induction temperature and pH were optimized in a 7-L bioreactor. The induction temperature and pH were set in the range of 24 to 30°C, and 4.0 to 7.0, respectively. The enzyme activity, total protein concentration, and WCW were detected throughout the fermentation.

### Purification, Kinetic Parameters, and Substrate Specificity

The recombinant *Sh*Csn46 was obtained by centrifuging fermented broth at 12,000 × *g* for 5 min at 6°C. Then, the obtained supernatant was concentrated by ultrafiltration with a membrane of 10 kDa cut-off. Finally, the recombinant *Sh*Csn46 was purified by Ni-IDA sefinose Resin chromatography (Sangon Biotech, Shanghai, China). The purified recombinant *Sh*Csn46 was analyzed by SDS-PAGE.

For substrate specificity, colloidal chitosan with different DDA (85, 90, and 95%), colloidal and powder chitin, xylan, soluble starch, and microcrystalline cellulose were used as substrate. The kinetic parameters were detected using different concentrations of colloidal chitosan with 95% DDA (1, 1.5, 2, 2.5, 3, 4, 5, 6, and 8 mg/ml) as substrate. The values of *K*_*m*_ and *V*_*max*_ were calculated by program Graft.

### Characterization of *Sh*Csn46

The optimal pH and pH stability of purified *Sh*Csn46 were detected according to the previous method (Qin et al., 2018). For optimal pH, the relative activity of purified *Sh*Csn46 was detected in different 50 mM buffer with pH in the range from 3.5 to 7.0 (HAc-NaAc for pH 3.5 to 6, Na_2_HPO_4_-NaH_2_PO_4_ for pH 6.5 to 7.0). The relative activity at different pH was calculated by setting that at pH 5.5 as 100%. The pH stability of purified *Sh*Csn46 was detected after being incubated at 30°C in 50 mM buffer with different pH from 4.0 to 11.0 for 6 h (HAc-NaAc for pH 3.0 to 6.0, Na_2_HPO_4_-NaH_2_PO_4_ for pH 6.0 to 8.0, Tris–HCl for pH 7.0 to 9.0, and Gly-NaOH for pH 9.0 to 10.0). The enzyme activity of *Sh*Csn46 treated with distilled water was considered as 100%. All measurements were carried out in triplicate.

For optimal temperature of purified *Sh*Csn46, the relative activities of purified *Sh*Csn46 at different temperatures in the range from 30 to 70°C were measured. The relative activities at different temperatures were calculated by setting that at 55°C as 100%. For thermal stability, the residual activity of purified *Sh*Csn46 was determined after incubation at temperature from 45 to 65°C for 30 and 60 min. The enzyme activity of purified *Sh*Csn46 without heat treatment was considered as 100%. All measurements were carried out in triplicate.

### Effects of Different Metal Ions on the Stability of *Sh*Csn46

The effects of different metal ions (Ca^2+^, Mg^2+^, Na^+^, K^+^, Li^+^, Zn^2+^, Mn^2+^, Co^2+^, Hg^2+^, Ag^+^, and Fe^2+^) on the stability of purified *Sh*Csn46 were analyzed by incubating enzyme samples for 4 h at 25°C. The enzyme activity of purified *Sh*Csn46 without metal ion was considered as 100%. All measurements were carried out in triplicate.

### Hydrolytic Pattern of *Sh*Csn46

The hydrolytic pattern of *Sh*Csn46 was analyzed based on previous works (Qin et al., 2018; Guo et al., 2019). Different COSs were used as substrate to analyze the hydrolytic pattern of *Sh*Csn46. Purified *Sh*Csn46 was added to 0.5% (w/v) COSs (dissolved in distilled water), then incubated at 55°C for 2 h. Samples withdrawn at different times (20, 40, 60, and 120 min) were immediately incubated at 90°C for 10 min. Thin-layer chromatography (TLC) method was used to analyze the samples withdrawn at different times. Samples were spotted on a TLC plate, developed in isopropanol/water/ammonium hydroxide (15:1:7.5, v/v) as solvent, and sprayed with 0.3% ninhydrin (dissolved in ethanol). The hydrolysis products were visualized by heating the plate at 100°C for 10 min.

### Preparation of Chitosan Oligosaccharides by *Sh*Csn46

The 4% (w/v) colloidal chitosan with 95% DDA and purified *Sh*Csn46 were used to prepare COSs with different DP. The 4% (w/v) colloidal chitosan was prepared as follows: 0.4 g chitosan was added to 10 ml HAc-NaAc buffer (pH 4.7, 0.2 mM), then preheated at 55°C and 100 rpm for 30 min, and finally incubated at 8°C for 4 h. Reactions were carried out in a 50-ml flask containing 10 ml 4% (w/v) colloidal chitosan with different amounts of *Sh*Csn46 (2, 5, 10, 15, 20, 25, and 30 U/ml) at 55°C and 100 rpm for 1 h. The reaction was stopped by incubating at 90°C for 10 min. For COS analysis, 5 ml hydrolysates of 4% colloidal chitosan was centrifuged at 10,000 × *g* for 5 min at 6°C and were analyzed by TLC and high-performance liquid chromatography (HPLC) method. The HPLC system (Thermo Fisher Scientific, Waltham, MA, United States) was equipped with a refractive index detector and a Zorbax carbohydrate analysis column (4.6 × 250 mm, 5 μm) (Agilent, Santa Clara, CA, United States). The mobile phase was composed of acetonitrile and water (70:30, v/v) and the flow rate was 1 ml/min. The concentrations of different COSs were quantified by integrating peak areas according to the respective standard curve (Qin et al., 2018). The effects of different *Sh*Csn46 additions on the production of the same COS were analyzed. Experiments were conducted in triplicate, and measurements were presented with their means and SD. Data were subjected to one-way ANOVA by SPSS (version 24.0) and Duncan’s multiple range tests (*p* < 0.05) to compare the mean value of different treatments. For hydrolysis rate analysis, 5-ml hydrolysates of 4% colloidal chitosan were added with 1 M NaOH to adjust the pH to 9.0, centrifuged at 10,000 × *g* for 5 min at 6°C, and then dried at 100°C. The method for calculation of hydrolysis rate is provided in Supplementary Material.

Furthermore, the hydrolytic process of 4% colloidal chitosan (w/v) addition with 10 U/ml of *Sh*Csn46 was investigated. The reaction was carried out in a 1,000-ml flask containing 200 ml 4% colloidal chitosan (w/v). The reaction samples (2 ml) were withdrawn at 5, 10, 15, 20, 25, 30, 40, and 50 min, respectively, and the methods for sample treatment and analysis were the same as previously mentioned. All measurements were carried out in triplicate.

## Results and Discussion

### Gene Cloning and Bioinformatics Analysis

Up to now, the complete genome sequences of two subspecies from *S. hygroscopicu*s have been sequenced, which include *S. hygroscopicus* XM201 and *S. hygroscopicus* KCTC1717. These two subspecies both contained hypothetical chitosanases from GH46 family. Therefore, two pairs of primers were designed for PCR amplification. The results of amplification revealed that only primers sh201-fw and sh201-rev could obtain a PCR product about 1,100 bp in length. The obtained PCR product was confirmed with the length of 1,103 bp by DNA sequencing. The results of NCBI-blastn exhibited that the obtained PCR product showed 97.5% identity to the sequences of the hypothetical chitosanase in the range from 3,191,731 to 3,192,597 of complete genome of *S. hygroscopicus* strain XM201. Based on the result of open reading frame finder, we deduced that the open reading frame of this gene (named *shcsn46*) was 867 bp, which encoded 288 amino acids. The sequence of *shcsn46* was deposited in the GenBank of NCBI (accession no. OL444888). The results of NCBI-blastp revealed that *Sh*Csn46 was a chitosanase, which shared 97.2% identity to a hypothetical chitosanase from *S. hygroscopicus* XM201. ProtParam analysis found that the molecular weight and theoretical p*I* of *Sh*Csn46 were 31.2 kDa and 5.51, respectively. Furthermore, the total number of negatively (Asp and Glu) and positively charged residues (Arg and Lys) were 42 and 34, respectively. SignalP 5.0 server analysis found that the first 31 amino acid residues of *Sh*Csn46 were signal peptides.

**FIGURE 1:**
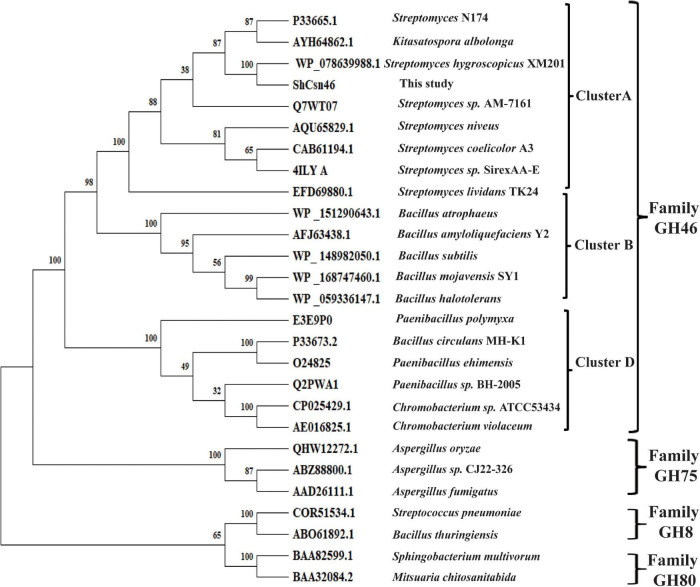
Phylogenetic analysis of *Sh*Csn46. Different chitosanases from GH8, GH46, GH75, and GH80 family were retrieved from GenBank.

According to the results of NCBI-blastp, we deduced that *Sh*Csn46 belonged to GH46 family. Meanwhile, a phylogenetic tree was constructed, which contained chitosanases from GH8, GH46, GH75, and GH80 family. As shown in Figure 1, *Sh*Csn46 is grouped to GH46 family and closely related to a hypothetical chitosanase from *S. hygroscopicus* XM201. It has been reported that the chitosanases from GH46 family can be divided into five subgroups (named A to E), and the chitosanases from A, B, and D are characterized more extensively than other subgroups (Viens et al., 2015). As depicted in Figure 1, *Sh*Csn46 belonged to subgroup A, which is mainly composed of the chitosanases from *Streptomyces*. Meanwhile, the alignment of *Sh*Csn46 against the already crystallized chitosanases of GH46 family was also investigated. Based on the results of alignment and previous studies (Marcotte et al., 1996; Saito et al., 1999; Takauka et al., 2014; Lyu et al., 2015), two amino acids (E41 and D59) were considered as catalytic active sites (Supplementary Figure 1).

**FIGURE 2:**
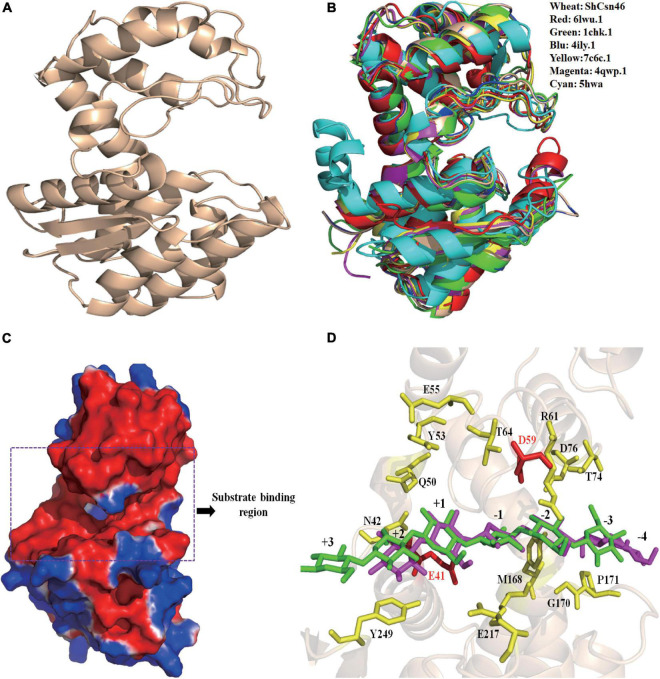
Homology-modeling structure of *Sh*Csn46. The overall structure of *Sh*Csn46 **(A)**. Structural alignment of *Sh*Csn46 with previously reported GH46 family chitosanases **(B)**. 4ily.1, 1chk.1, 4qwp.1, 6lwu.1, 5hwa, and 7c6c represent the tertiary structure of chitosanases from *Streptomyces* sp. SirexAA-E, *Streptomyces* sp. N174, *Microbacterium* sp., *Gynuella sunshinyii* YC6258, *Bacillus circulans* MH-K1, and *Bacillus subtilis* MY002, respectively. Surface electrostatic potential of *Sh*Csn46 **(C)**. The surface coloring is based on the electrostatic potential with a gradient from red (electronegative) to blue (electropositive). Interactions between (GlcN)_6_ and *Sh*Csn46 **(D)**. The amino acids residues E41 and D59 colored in red are catalytic active sites; the amino acids residues colored in yellow play an important role in binding the substrate.

The tertiary structure of *Sh*Csn46 was obtained by homology-modeling using the chitosanases from *Streptomyces* sp. N174 as template (PDB deposition: 1chk.1). The tertiary structure of *Sh*Csn46 can be divided into upper and lower lobes, which was mainly composed of α-helices (Figure 2A). Although the identities of amino acids from already crystallized chitosanases were very low (Supplementary Figure 1), the tertiary structures of these chitosanases were similar (Figure 2B). As shown in Figure 2C, the substrate binding region of *Sh*Csn46 is highly negatively charged, which is suitable to bind the cationic polysaccharide chitosan. Besides, the substrate binding region of *Sh*Csn46 was an open cleft (Figure 2C), which is similar to the chitosanases from *Streptomyces* sp. SirexAA-E, *Streptomyces* sp. N174, *Microbacterium* sp., and *Bacillus circulans* MH-K1 (Marcotte et al., 1996; Saito et al., 1999; Takauka et al., 2014; Lyu et al., 2015). However, Li et al. (2021) demonstrated that the substrate binding region of the chitosanase CsnMY002 from *Bacillus subtilis* MY002 is a closed tunnel, which means different chitosanases with different transition mechanisms. The hydrogen bonds were the main force to stabilize the network between (GlcN)_6_ and substrate binding region of *Sh*Csn46. Based on the results of previous studies (Marcotte et al., 1996; Saito et al., 1999; Takauka et al., 2014; Lyu et al., 2015; Li et al., 2021), we deduced that several residues (Tyr29, Arg37, Ala44, Gly45, Thr50, Asp52, Tyr118, Gln146, Asp149, Trp204, Glu206, and Glu235) play an important role in binding the substrate (Figure 2D).

### Overexpression of *Sh*Csn46 in *Pichia pastoris* X33

Previous studies demonstrated that codon usage plays an important role in the expression level of heterologous protein in *P. pastoris* (Yang and Zhang, 2018). Analysis of the sequence of *shcsn46* found that the GC content is 67%, which is far above the appropriate range for optimal expression in yeast. Furthermore, some codons rarely used in *P. pastoris* such as CCG (Pro), CTC (Leu), and CGG (Arg) presented in *shcsn46*. Therefore, the sequence of *shcsn46* without signal sequence (named *shcsn46s*) was optimized according to the codon usage of *P. pastoris*. The GC content was adjusted from 67 to 47%. The rarely used codons were replaced by the most frequently used codons. In total, 179 nucleotides were optimized, and the optimized gene (*shcsn46s-opt*) showed 76.7% identity to the native gene (Supplementary Figure 2).

The optimized gene (*shcsn46s-opt*) was synthesized, ligated into pPICZαA, and transformed into *P. pastoris* X33. After transformation, many colonies were formed on the YPDZ plates. In the process of preliminary screening, four recombinant strains (named X33-Sh12, X33-Sh33, X33-Sh69, and X33-Sh83) with higher activity were isolated from 96 colonies. The chitosanase activities of X33-Sh12, X33-Sh33, X33-Sh69, and X33-Sh83 were 7.2, 8.5, 7.5, and 6.8 U/ml, respectively. Furthermore, those four recombinant strains were cultivated in a shake flask, and the results are shown in Supplementary Figure 3. The recombinant strain X33-Sh33 exhibited the highest activity (31.5 U/ml), followed by X33-Sh12 (28.5 U/ml) and X33-Sh69 (27.3 U/ml). Therefore, the recombinant strain X33-Sh33 was chosen for high cell density fermentation.

**FIGURE 3:**
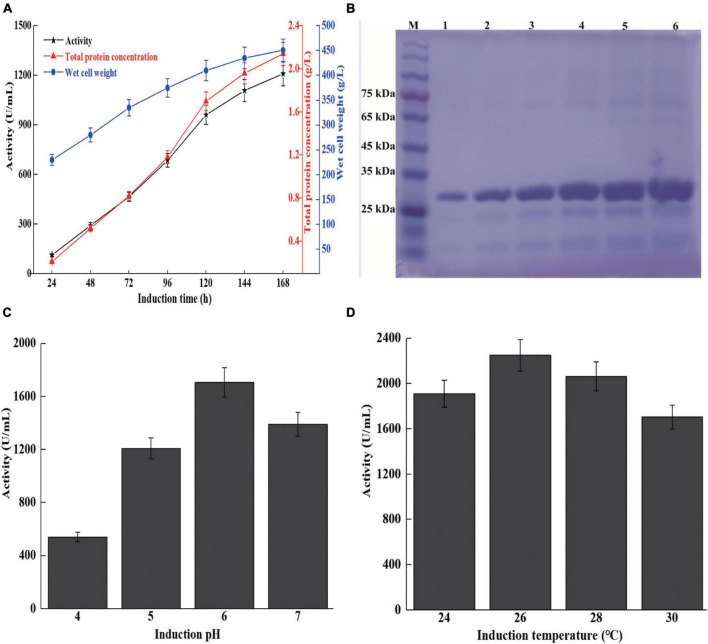
High cell density fermentation of recombinant strain X33-Sh33 in 7-L bioreactor. The chitosanase activity, total protein concentration, and WCW during fed-batch fermentation in 7-L bioreactor **(A)**. SDS-PAGE analysis of supernatants from different induction times **(B)**. M represents marker, lanes 1–6 represent supernatant from 48 to 168 h. Influence of induction pH **(C)** and temperature **(D)** on the production of *Sh*Csn46.

High cell density fermentation was carried out in a 7-L bioreactor. The maximum activity and total protein concentration of recombinant strain X33-Sh33 cultivated in a 7-L bioreactor were 1,209 U/ml and 2.15 g/l, respectively (Figure 3A). Meanwhile, the supernatant from different induction times were analyzed by SDS-PAGE. The *Sh*Csn46 exhibited one band corresponding to 28 kDa and was the main protein of supernatant regardless of the induction time (Figure 3B). High cell density fermentation is an effective method to improve the production of chitosanase. The production of recombinant chitosanase from *Aspergillus fumigatus* CJ22-326 (*A. fumigatus* CJ22-326) is almost improved by sevenfold from shake flask to 5-L bioreactor (Zhou et al., 2020). Meanwhile, the expression level of recombinant chitosanase from *Bacillus amyloliquefaciens* (*B. amyloliquefaciens*) is almost improved by 35.91-fold by high cell density fermentation (Luo et al., 2020). The results of this study are similar to previous works; the maximum activity of the recombinant strain X33-Sh33 in 7-L bioreactor was 1209 U/ml, which was 38.3-fold higher than that in shake flask.

Induction pH and temperature play an important role in the production of recombinant protein in *P. pastoris*. Previous studies demonstrated that optimization of induction pH and temperature could improve the production of recombinant protein (Ahmad et al., 2014; Yang and Zhang, 2018). The induction pH and temperature of high cell density fermentation were optimized. As shown in Figure 3C, the activities of induction pH at 4.0 to 7.0 are 541, 1209, 1705, and 1410 U/ml, respectively. Besides, we found that lower induction temperature was beneficial for the expression of *Sh*Csn46. The maximum chitosanase activity at 26 and 28°C reached 2,250 and 2,063 U/ml, respectively, which were 1.32- and 1.21-fold higher than that at 30°C (Figure 3D). The result of this study is similar to previous works (Wang et al., 2009, 2019). For instance, the production of *Thermomyces dupontii* lipase and *Bacillus* sp. alkaline polygalacturonate lyase are improved by 1.4- and 2.1-fold, respectively, after optimization of induction pH and temperature (Wang et al., 2009, 2019).

According to the previous studies, *E. coli* is the most used host for expression of different chitosanases due to its easy cultivation, short growth times, and mature process for purification (Huang et al., 2016; Zheng et al., 2021). Nevertheless, intracellular expression and inclusion bodies are the major obstacles limiting *E. coli* as a host for large-scale preparation of recombinant chitosanase. Compared with *E. coli*, *P. pastoris* has many advantages such as extracellular expression, powerful secretion ability, and mature fermentation process (Ahmad et al., 2014; Yang and Zhang, 2018). Therefore, *P. pastoris* is more suitable for large-scale production of recombinant chitosanase than *E. coli*. Several chitosanases have been heterologously expressed in *P. pastoris*, and the results of these researches demonstrated that *P. pastoris* exhibits its potential for large-scale preparation of recombinant chitosanases. The expression level of chitosanases from *Mitsuaria* sp., *Streptomyces* sp. N174, *B. amyloliquefaciens*, *A. fumigatus* CJ22-326, and *Bacillus mojavensis* SY1 (*B. mojavensis* SY1) are almost 1.6, 8.5, 4.5, 3.1, and 3.8 g/l, respectively (Peng et al., 2013; Ding et al., 2019; Luo et al., 2020; Zhou et al., 2020; Wang J. R. et al., 2021). Similar with previous works, the production of *Sh*Csn46 was 3.91 g/l and the recombinant *Sh*Csn46 was the main protein of supernatant (Figure 3B).

### Purification, Kinetic Parameters, and Substrate Specificity

The recombinant *Sh*Csn46 was purified from supernatant and the specific activity of purified *Sh*Csn46 was 988 U/mg (Supplementary Figure 4 and Supplementary Table 1). The values of *K*_*m*_, *V*_*max*_, and *K*_*cat*_ of purified *Sh*Csn46 were 2.1 mg/ml, 959 μM min^–1^ ml^–1^, and 206.8 min^–1^, respectively (Table 1). Kinetic parameters of purified *Sh*Csn46 revealed that it has high substrate affinity and catalytic efficiency toward chitosan. Analyzing previous studies, we found that *K*_*m*_ values of *Streptomyces* chitosanases vary differently. The *K*_*m*_ values of chitosanases from *Streptomyces albolongus* ATCC27414 (*S. albolongus* ATCC27414), *Streptomyces niveus* (*S. niveus*), *Streptomyces avermitilis* (*S. avermitilis*), *Streptomyces* sp. SirexAA-E, and *Streptomyces* sp. N174 are 7.4, 1.8, 1.3, 2.2, and 0.026 mg/ml, respectively (Lacombe-Harvey et al., 2013; Takauka et al., 2014; Guo et al., 2019, 2021; Chen et al., 2021).

**TABLE 1:** The kinetic parameters of *Sh*Csn46.

Kinetic parameters	Value
*K*_*m*_ (mg/ml)	2.1
*V*_*max*_ (μM min^–1^ ml^–1^)	959
*K*_*cat*_ (min^–1^)	206.8
*K*_*cat*_/*K*_*m*_ (ml mg^–1^ min^–1^)	98.5

The results of substrate specificity of purified *Sh*Csn46 are shown in Table 2. Purified *Sh*Csn46 exhibited the highest activity toward colloidal chitosan with 95% DDA, followed by colloidal chitosan with 90 and 85% DDA, respectively (Table 2). Meanwhile, purified *Sh*Csn46 exhibited no activity toward microcrystalline cellulose, xylan, soluble starch, powder, and colloidal chitin. The activity of chitosanase from GH46 family is relative to the deacetylation of chitosan, and many chitosanases show higher activity toward colloidal chitosan with 95% DDA than 85% DDA (Sun et al., 2020; Yang et al., 2020). Furthermore, chitosanases from GH46 family exhibits no activity toward many polysaccharides except chitosan. In contrast with GH46 family, chitosanases from GH5 and GH8 family exhibit activity toward sodium carboxymethylcellulose and β-glucan (Jiang et al., 2021; Wang Y. X. et al., 2021).

**TABLE 2:** The substrate specificity of *Sh*Csn46.

Substrates	Residual activity (%)
Colloidal chitosan with 85% DDA	89
Colloidal chitosan with 90% DDA	93
Colloidal chitosan with 95% DDA	100
Microcrystalline cellulose	ND*
Colloidal chitin	ND
Powder chitin	ND
Soluble starch	ND
Xylan	ND

*ND represents the enzyme activity was not detected.

### Characterization of *Sh*Csn46

**FIGURE 4:**
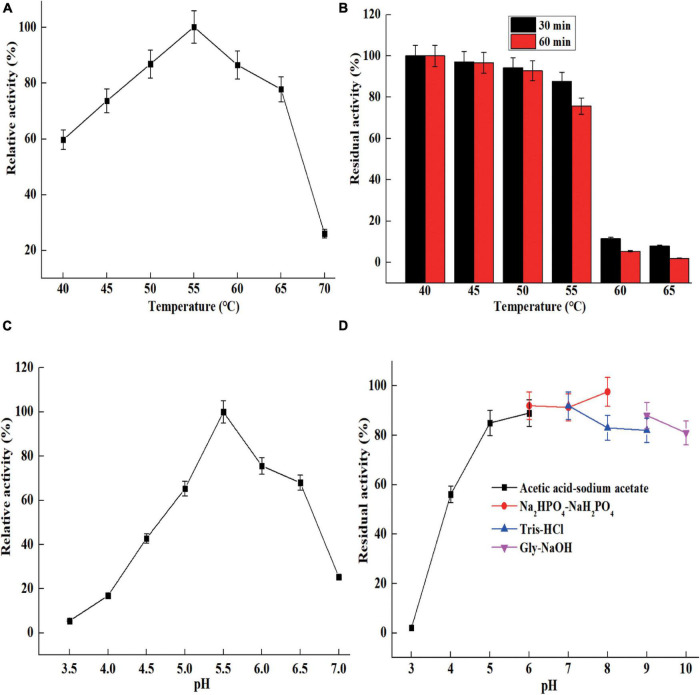
The characterization of purified *Sh*Csn46. Optimum temperature **(A)**, thermal stability **(B)**, optimum pH **(C)**, and pH stability **(D)**.

The optimal temperature of *Sh*Csn46 was 55°C and the relative activities were above 70% at the range from 45 to 65°C (Figure 4A). The results of thermal stability revealed that *Sh*Csn46 was stable from 40 to 55°C. The residual activity of 50, 55, and 60°C were 92.1, 75.3, and 23.2%, respectively, after heat treatment for 60 min (Figure 4B). Analyzing chitosanases from different *Streptomyces*, we found that the optimal temperatures of most reported *Streptomyces* chitosanases are 50°C. However, the thermal stability varies differently between *Streptomyces* chitosanases. The chitosanase Sn-CSN from *S. niveus* is stable below 35°C (Chen et al., 2021). Otherwise, the chitosanase from *Streptomyces roseolus* (*S. roseolus*) shows excellent thermal stability and the residual activity is above 90% after treatment at 60°C for 30 min (Jiang et al., 2012). Besides, the chitosanase SsCsn46 from *Streptomyces* sp. N174 retained more than 40% of maximum activity after 120 min of treatment at 50°C (Ding et al., 2019). The *Sh*Csn46 was active and stable from 45 to 55°C and exhibited better temperature property than some *Streptomyces* chitosanases. Furthermore, the temperature properties of *Sh*Csn46 revealed that it is suitable for preparation of COSs at 55°C. Reaction at high temperature is helpful for improving hydrolysis efficiency and reducing risk of microbial contamination.

The optimal pH of *Sh*Csn46 was 5.5 and the relative activities were above 60% at the range from pH 5.0 to 6.5 (Figure 4C). It has been reported that most of the chitosanases from *Streptomyces* are active from acidic to neutral range. The chitosanases from *S. avermitilis*, *S. roseolus*, and *Streptomyces* sp. N174 exhibit maximum activity at pH 5.0, 6.0, and 6.5, respectively (Jiang et al., 2012; Ding et al., 2019; Guo et al., 2021). However, the optimal pH of chitosanase Csn21c from *S. albolongus* ATCC27414 is 8.0 (Guo et al., 2019). Normally, chitosanases with maximum catalytic activity at acidic condition is helpful for preparation of COSs because chitosan shows better solubility below pH 6.0, and the solubility of chitosan is a key factor on efficiency of hydrolysis. As shown in Figure 4D, *Sh*Csn46 is stable from pH 5.0 to 9.0, and the residual activity is above 75% after 6 h of incubation at 25°C (Figure 4D). Similar to thermal stability, the pH stability of different *Streptomyces* chitosanases also varies greatly. The chitosanase Csn21c from *S. albolongus* ATCC is stable from pH 7.0 to 10.0 (Guo et al., 2019). Different from Csn21c, the chitosanase from *S. roseolus* is stable from pH 5.0 to 7.0 and the residual activity decreased sharply when reaction pH is above 7.0 (Jiang et al., 2012). Furthermore, the chitosanase Sn1-CSN from *S. niveus* is stable from pH 4.0 to 11.0 and the residual activity is above 80% even when the incubation time is 120 h (Chen et al., 2021). In this study, *Sh*Csn46 is active and stable from pH 4.5 to 6.0, which is suitable for preparation of COSs.

### Effects of Different Metal Ions on the Stability of *Sh*Csn46

*Sh*Csn46 was activated by Mn^2+^ and Mg^2+^, and the residual activities of *Sh*Csn46 were 123 and 135%, respectively, in the presence of 1 and 5 mM Mn^2+^ (Table 3). Besides, *Sh*Csn46 was inhibited by Cu^2+^, Fe^2+^, and Al^3+^. The residual activities of *Sh*Csn46 treated with 5 mM Cu^2+^, Fe^2+^, and Al^3+^ were only 8, 3, and 6%, respectively. The result of this study is similar to previous researches; the chitosanases from *S. roseolus* and *S. avermitilis* are activated by Mg^2+^ and inhibited by Cu^2+^ (Jiang et al., 2012; Guo et al., 2021).

**TABLE 3:** Effects of different metal cations on *Sh*Csn46 stability.

Metal ions	Residual activity (%)	
	1 mM	5 mM
Na^+^	97	93
K^+^	98	96
Cu^2+^	18	8
Mn^2+^	123	135
Mg^2+^	113	105
Fe^2+^	5	3
Co^2+^	98	93
Al^3+^	12	6
Zn^2+^	96	91
Ca^2+^	98	95

### Hydrolytic Pattern of *Sh*Csn46

**FIGURE 5:**
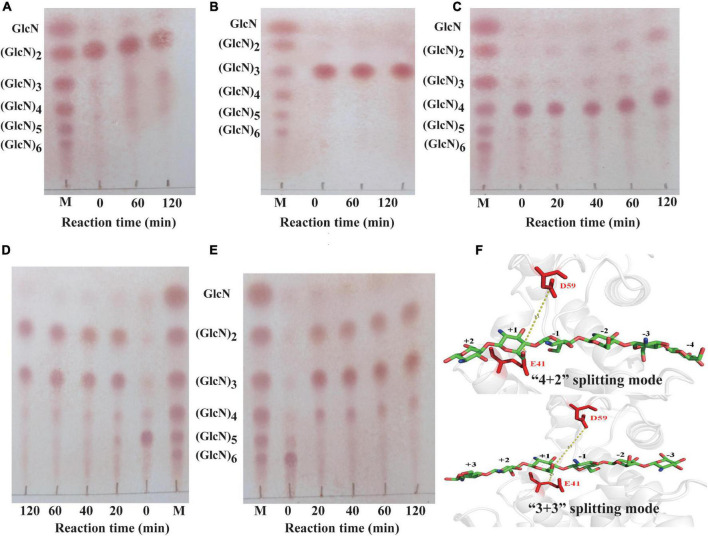
Analysis of the hydrolytic pattern of *Sh*Csn46 toward different COSs. Chitobiose **(A)**, chitotriose **(B)**, chitotetraose **(C)**, chitopentaose **(D)**, chitohexaose **(E)**. M, standards sugars containing GlcN, (GlcN)_2_, (GlcN)_3_, (GlcN)_4_, (GlcN)_5_, and (GlcN)_6_. **(F)** The “4 + 2” and “3 + 3” splitting mode of ShCsn46 toward (GlcN)_6_. The amino acids residues E41 and D59 colored in red are catalytic active sites.

The hydrolytic pattern of *Sh*Csn46 was investigated using (GlcN)_2_, (GlcN)_3_, (GlcN)_4_, (GlcN)_5_, and (GlcN)_6_ as substrates. As shown in Figures 5A,B, *Sh*Csn46 shows no activity toward (GlcN)_2_ and (GlcN)_3_; no products smaller than (GlcN)_2_ and (GlcN)_3_ were detected even after 2 h of reaction. Besides, *Sh*Csn46 exhibited little activity toward (GlcN)_4_. Little (GlcN)_4_ was hydrolyzed and converted to (GlcN)_2_ after 2 h of reaction (Figure 5C). Furthermore, *Sh*Csn46 displayed high activity toward (GlcN)_5_ and (GlcN)_6_. Most of (GlcN)_5_ was cleaved and converted to (GlcN)_2_ and (GlcN)_3_ after 20-min reaction (Figure 5D). The (GlcN)_6_ was completely cleaved and transformed to (GlcN)_2_, (GlcN)_3_, and (GlcN)_4_ when reaction time was 20 min (Figure 5E). Analyzing the results of different COSs hydrolyzed by *Sh*Csn46, we found that no GlcN was detected, which means *Sh*Csn46 is an endo-type chitosanase. Besides, no COSs with higher DP than the corresponding substrates were detected, which suggests *Sh*Csn46 without transglycosylation activity.

In this study, *Sh*Csn46 hardly hydrolyzed the β-1,4 linkage in (GlcN)_4_, suggesting *Sh*Csn46 has great potential to prepare COSs with higher DP. Meanwhile, the hydrolytic pattern of *Sh*Csn46 is similar to many previous reported chitosanases. Most of the chitosanases from GH46 display endo-type property and without activity toward (GlcN)_2_ and (GlcN)_3_ (Jiang et al., 2012; Luo et al., 2020; Sun et al., 2020; Zhou et al., 2020; Wang J. R. et al., 2021). Furthermore, the hydrolytic process of (GlcN)_6_ revealed that *Sh*Csn46 may have two binding and cutting modes, which are “4 + 2” and “3 + 3” splitting mode, respectively (Figure 5F). During the process of “4 + 2” splitting mode, (GlcN)_6_ was hydrolyzed and converted to (GlcN)_2_ and (GlcN)_4_. Meanwhile, in the “3 + 3” splitting mode (GlcN)_6_, was hydrolyzed and converted to (GlcN)_3_.

### Preparation of Chitosan Oligosaccharides by *Sh*Csn46

**FIGURE 6:**
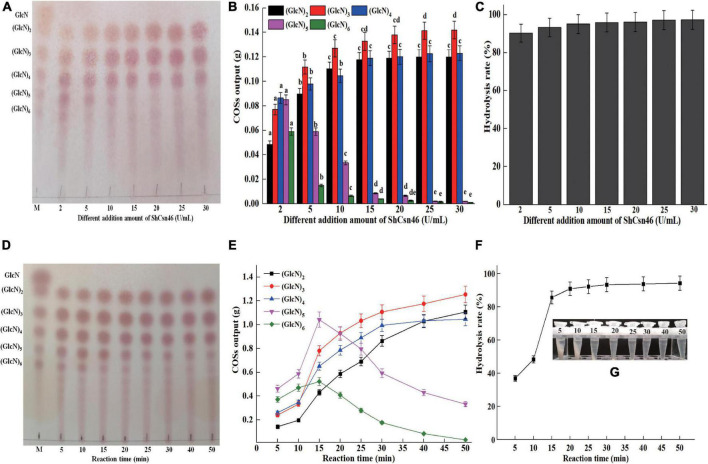
Preparation of COSs by *Sh*Csn46. **(A)** TLC analysis of hydrolysates from 4% (w/v) colloidal chitosan addition with different amounts of *Sh*Csn46. **(B)** HPLC analysis of COSs output from different hydrolysates. The effects of different *Sh*Csn46 additions on the production of the same COS were analyzed. Different lowercase superscripts in the columns with the same color indicate statistical difference (*p* < 0.05). **(C)** The hydrolysis rate of 4% colloidal chitosan addition with different amounts of *Sh*Csn46. **(D)** TLC analysis of hydrolysates from 4% colloidal chitosan addition with 10 U/ml purified *Sh*Csn46 at different reaction times. **(E)** HPLC analysis of COSs output from 4% colloidal chitosan addition with 10 U/ml purified *Sh*Csn46 at different reaction times. **(F)** The hydrolysis rates of 4% colloidal chitosan with 10 U/ml purified *Sh*Csn46 at different reaction times. **(G)** The precipitation of undegraded chitosan with 10 U/ml purified *Sh*Csn46 at different reaction times.

The 4% (w/v) colloidal chitosan with 95% DDA and purified *Sh*Csn46 were used for the preparation of COSs. Initially, different additions of *Sh*Csn46 (2 to 30 U/ml) were investigated. As shown in Figures 6A,B, the hydrolysates of 4% colloidal chitosan mainly include (GlcN)_2_, (GlcN)_3_, (GlcN)_4_, (GlcN)_5_, and (GlcN)_6_, when the amount of *Sh*Csn46 was 2 U/ml. As the amount of *Sh*Csn46 increased to 5 and 10 U/ml, the hydrolysates were mainly composed of (GlcN)_2_, (GlcN)_3_, (GlcN)_4_, and (GlcN)_5_. With the increasing addition of *Sh*Csn46 to 15, 20, 25, and 30 U/ml, the end products were mainly composed of (GlcN)_2_, (GlcN)_3_, and (GlcN)_4_ (Figures 6A,B). The hydrolysis rates of all reactions were above 90.3% (Figure 6C). Based on the results of initial preparation of COSs, the composition of different COSs and hydrolysis rate at different reaction times were further studied. As depicted in Figures 6D,E, the hydrolysates mainly include (GlcN)_2_, (GlcN)_3_, (GlcN)_4_, (GlcN)_5_, and (GlcN)_6_ after 5, 10, and 15 min of reaction. As the reaction time increased to 20, 25, and 30 min, the COS mixture was mainly composed of (GlcN)_2_, (GlcN)_3_, (GlcN)_4_, and (GlcN)_5_. For reaction time that reached 40 and 50 min, the end products were mainly composed of (GlcN)_2_, (GlcN)_3_, and (GlcN)_4_ (Figures 6D,E). As shown in Figure 6F, the hydrolysis rates are 37.2 and 48.6%, respectively, when the reaction times are 5 and 10 min. As reaction time increased to 15 min, the hydrolysis rate was 86.1%. The hydrolysis rates of reaction time from 20 to 50 min were in the range from 91.2 to 95.3% (Figures 6F,G).

Recently, non-specific enzymes and chitosanases from different GH families have been used to produce COSs (Table 4). As shown in Table 4, the chitosanases are more efficient than non-specific enzymes for preparation of COSs. The chitosanase PbCsn8, which belonged to GH8 family, from *Paenibacillus barengoltzii* could hydrolyze 79.3 of 5% colloidal chitosan into (GlcN)_2_, (GlcN)_3_, and (GlcN)_4_ after 4-h reaction in the presence of 5 U/ml of PbCsn8 (Jiang et al., 2021). As a member of the GH75 family, the chitosanase from *A. fumigatus* CJ22-326 could hydrolyze 2% colloidal chitosan to produce COSs with different DP (2–6) and the hydrolysis rate is 97.29% after 8 h of reaction in the presence of 30 U/ml of this chitosanase (Zhou et al., 2020). As shown in Table 4, the GH46 family chitosanases BaCsn46A, Csn-SH, and CsnBm from *B. amyloliquefaciens*, *B. atrophaeus* BSS, and *B. mojavensis* SY1 exhibit higher efficiency toward colloidal than non-specific enzymes and chitosanase from GH8 and GH75 family (Qin et al., 2018; Cui et al., 2021; Wang J. R. et al., 2021). In this study, after 20 min of reaction in the presence of 10 U/ml *Sh*Csn46, the hydrolysis rate of 4% colloidal chitosan reached 91.2%, which reveals that *Sh*Csn46 has great potential and competitiveness for preparation of COSs. Furthermore, *Sh*Csn46 is suitable to prepare COSs with desirable DP by controllable reaction.

**TABLE 4:** Enzymatic conversion of chitosan to COSs.

Enzyme	Amount of enzyme	Chitosan	Reaction time (h)	Hydrolysis rates (%)	DP of major products	References
*Sh*Csn46	10 U/ml (about 1%)	4% with 95% DDA	0.33	91.2	2–6	This study
Chitosanase from *A. fumigatus* CJ22-326	30 U/ml	2% with 95% DDA	8	97.3	2–6	Zhou et al., 2020
PbCsn8	5 U/ml	5 with 85% DDA	4	79.3	2–4	Jiang et al., 2021
BaCsn46A	3 U/ml	3 with 95% DDA	3	86.9	2–6	Qin et al., 2018
Csn-SH	20 U/ml	4% with 85% DDA	0.67	80.6	2–6	Cui et al., 2021
CsnBm	9 U/ml	4% with 90% DDA	0.25	92.3	2–6	Wang J. R. et al., 2021
Commercial pectinase	10%	1% with 88% DDA	24	17.0	6–11	Cabrera and Van Cutsem, 2005
Commercial lipase	0.5%	0.5% with 83% DDA	6	58.2	1–6	Lee et al., 2008
Commercial papain	0.3%	1% with 87% DDA	24	11.1	3–7	Lin et al., 2002
Commercial cellulase	0.25%	0.5% with 87% DDA	24	40.0	2–6	Tegl et al., 2016

## Conclusion

In conclusion, a chitosanase (*Sh*Csn46) from *S. hygroscopicus* R1 was bioinformatics analyzed, overexpressed, purified, and characterized. *Sh*Csn46 belonged to subgroup A of the GH46 family. The maximum activity of *Sh*Csn46 was 2250 U/ml. The purified *Sh*Csn46 was most active at 55°C and pH 5.5. In addition, *Sh*Csn46 is an endo-type chitosanase and exhibited high efficiency toward 4% colloidal chitosan to produce COSs with desirable DP. The excellent properties and overexpression of *Sh*Csn46 will provide a basis for its application in preparation of COSs.

## Data Availability Statement

The datasets presented in this study can be found in online repositories. The names of the repository/repositories and accession number(s) can be found below: https://www.ncbi.nlm.nih.gov/genbank/, OL444888.

## Author Contributions

JW contributed to constructing recombinant strain and bioinformatics analysis of *Sh*Csn46. PW contributed to high cell density fermentation. WC and SY contributed to analysis of chitosanase activity. MZ and BZ contributed to analysis of COSs by TLC and HPLC. All authors contributed to the article and approved the submitted version.

## Conflict of Interest

JW, PW, MZ, WC, SY, and BZ are employed by Shenzhen Raink Ecology & Environment Co., Ltd.

## Publisher’s Note

All claims expressed in this article are solely those of the authors and do not necessarily represent those of their affiliated organizations, or those of the publisher, the editors and the reviewers. Any product that may be evaluated in this article, or claim that may be made by its manufacturer, is not guaranteed or endorsed by the publisher.

## Footnotes

1
https://www.thermofisher.com
