# Supplementary material for: Overexpression and Biochemical Properties of a GH46 Chitosanase From Marine Streptomyces hygroscopicus R1 Suitable for Chitosan Oligosaccharides Preparation
Source: Front Microbiol. 2022 Jan 31;12:816845. doi: 10.3389/fmicb.2021.816845 (PMC8841797; doi:10.3389/fmicb.2021.816845)
Supplement: Supplementary file 1 [file Data_Sheet_1.docx]

Supplementary Material

# Supplementary Methods

**Protocol for Selection of Recombinant Strains**

The transformants were picked and cultured in 24-deep-well micro-plates containing 1.8 mL/well BMGY medium at 30 ºC for 24 h. After this, the cells were harvested by centrifugation, re-suspended, and cultured in 1.6 mL/well BMMY medium. After 24 h, plates were subjected to centrifugation again and supernatants were used in subsequent activity assays. The clones showing higher activities were checked by shaking flask fermentation.

**Shake Flask Cultures**

The single-copied recombinant strains were inoculated into 10 ml BMGY in a 150 mL flask and incubated at 30 °C and 200 rpm for 24 h. Then the recombinant cells were harvested by centrifugation, re-suspended in BMMY, and transferred to 50 mL BMMY (OD600 is 1.0) in a 250 mL flask, incubated at 30 °C and 200 rpm. 0.75% (v/v) methanol was added to the culture at every 24 h, and 1 mL culture was harvested every 24 h for chitosanase assay.

**Protocol for High Cell Density Fermentation**

Inoculum was cultured in BMGY medium. Cells were grown for 18-20 h at 30 °C on shaker of 200 rpm. Then, 10 % (v/v) of the inoculum was inoculated into the 7 and 50 L bioreactors containing 2.5 and 20 L basal salt medium, which made of 0.47 g/L CaSO4•2H2O, 9.1 g/L K2SO4, 7.5 g/L MgSO4•7H2O, 6.2 g/L KOH, 13.35 ml/L H3PO4 (85%), 20.0 g/L glycerol and 1.5 mL Pichia trace metal 1 (PTM1). One liter PTM1 consists of 6 g CuSO4•5H2O, 0.08 g NaI, 3 g MnSO4•H2O, 0.5 g CoCl2, 20 g ZnCl2, 0.02 g H3BO3 , 0.2 g Na2MnO4•2H2O, 65 g FeSO4•7H2O, 0.2 g biotin and 30 ml 6 N H2SO4. The temperature was controlled at 30 °C and the pH was maintained at 5.0 using NH4OH (28 %) and H3PO4 (10 %). For 7 L bioreactor, the agitation rate was set at 600 rpm and the aeration rate was 30 L/min. For 50 L bioreactor, the agitation rate was set at 500 rpm and the aeration rate was 40 L/min. When glycerol was used up, as indicated by an increased in dissolved oxygen (DO), 0.5 % (v/v) methanol was added to induce expression lipase. Feeding of methanol was linked to the dissolved oxygen (DO). When the initial methanol 0.5 % (v/v) was depleted (indicated by an abrupt increase in DO), 100% methanol solution containing of 1.2 % (v/v) PTM1 was added automatically. The concentration of methanol was kept stable by monitoring the dissolved oxygen (OD) content and maintaining it at greater than 20 %. The enzyme activity of the supernatant and dry cell weight were monitored throughout the cultivation.

**Calculation of Hydrolysis rate**

The hydrolysis rate was calculated according to the following formula.


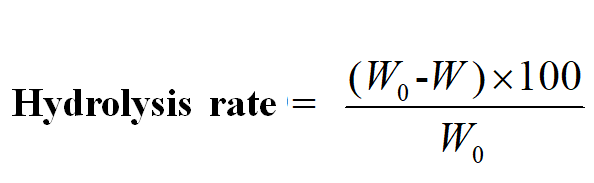


*W*_0_ and *W* refer to the average dry weight of chitosan precipitated in the control and experimental groups, respectively.

# Supplementary Figures and Tables

# Supplementary Table1 Purification of ShCsn46 from culture supernatant

| Purification steps | Total activity (U) | Total protein  (mg) | Specific activity (U/mg) | Purification fold |
| --- | --- | --- | --- | --- |
| Supernatant | 141865 | 251 | 565 | 1 |
| Ultrafiltration | 111870 | 165 | 678 | 1.18 |
| Affinity chromatography | 81016 | 82 | 988 | 1.75 |


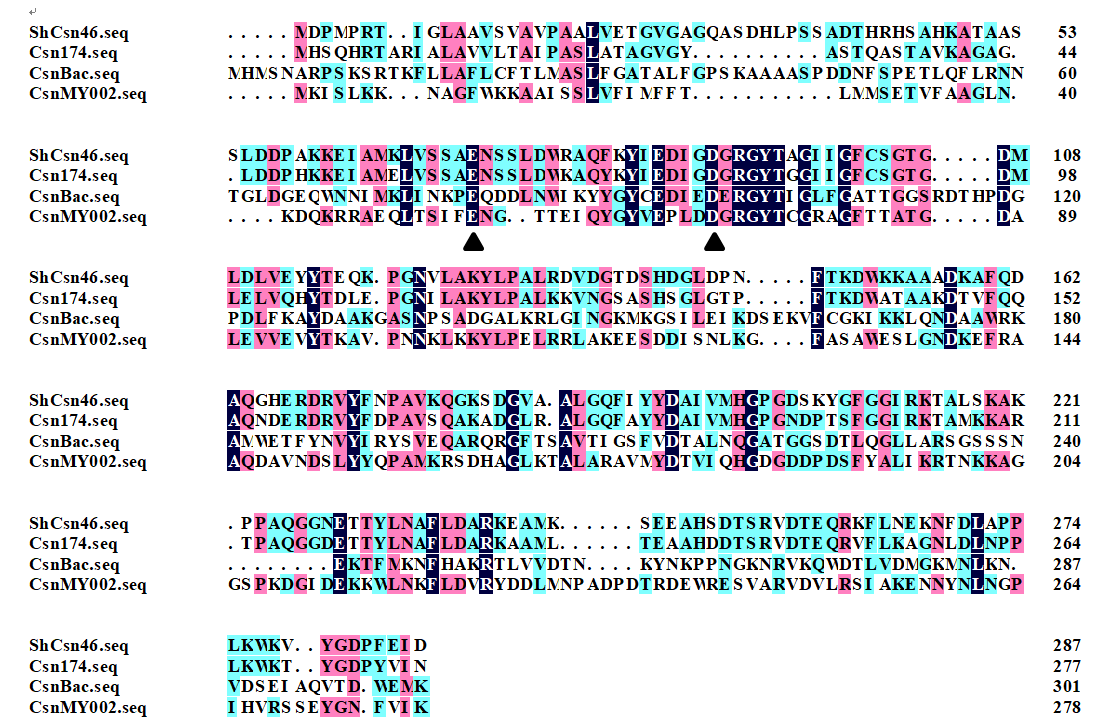


Supplementary Figure 1 Multiple amino acid sequence alignment of ShCsn46 with other previously reported GH family 46 chitosanases.

Supplementary Figure 2 Sequence comparison between the native (*shcsn46*) and optimized gene (*shcsn46-opt*).


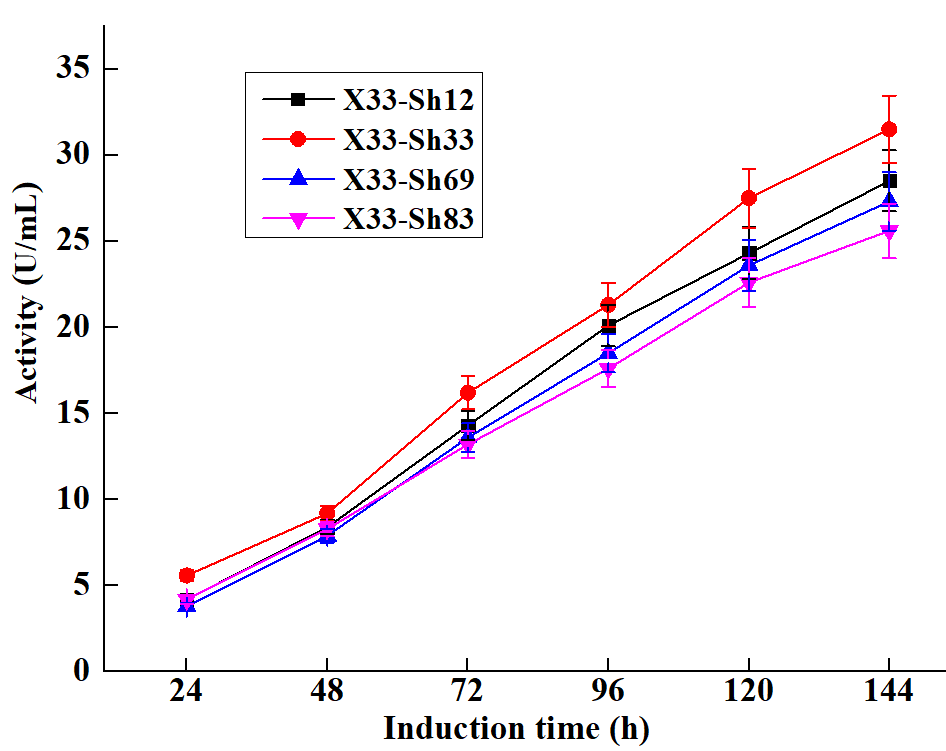


Supplementary Figure 3 The chitosanase activity of different recombinant strains in shake flask cultures.

#
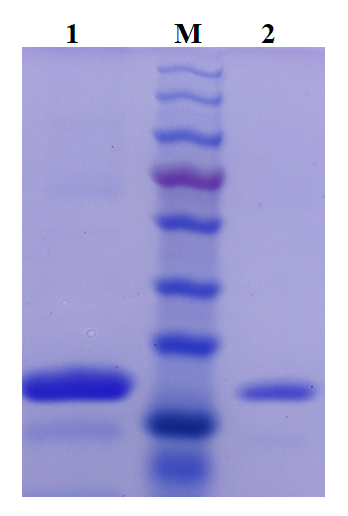


Supplementary Figure 4 SDS-PAGE analysis of purified ShCsn46. M: marker, lane 1: supernatant,

lane 2: purified ShCsn46.
